# From Street Canyons to Corridors: Adapting Urban Propagation Models for an Indoor IQRF Network

**DOI:** 10.3390/s25226950

**Published:** 2025-11-13

**Authors:** Talip Eren Doyan, Bengisu Yalcinkaya, Deren Dogan, Yaser Dalveren, Mohammad Derawi

**Affiliations:** 1 Department of Electrical and Electronics Engineering, Atilim University, Ankara 06830, Turkey; doyan.taliperen@student.atilim.edu.tr (T.E.D.); bengisu.yalcinkaya@atilim.edu.tr (B.Y.); 2 Department of Computer Engineering, Izmir Bakircay University, Izmir 35665, Turkey; 3 School of Telecommunications Engineering, Universitat Politècnica de València, 46022 Valencia, Spain; ddogan@teleco.upv.es; 4 Department of Electrical and Electronics Engineering, Izmir Bakircay University, Izmir 35665, Turkey; yaser.dalveren@bakircay.edu.tr; 5 Department of Electronic Systems, Norwegian University of Science and Technology, 2815 Gjovik, Norway

**Keywords:** internet of things, smart buildings, IQRF, indoor propagation modeling, diffraction, site-specific model

## Abstract

Among wireless communication technologies underlying Internet of Things (IoT)-based smart buildings, IQRF (Intelligent Connectivity Using Radio Frequency) technology is a promising candidate due to its low power consumption, cost-effectiveness, and wide coverage. However, effectively modeling the propagation characteristics of IQRF in complex indoor environments for simple and accurate network deployment remains challenging, as architectural elements like walls and corners cause substantial signal attenuation and unpredictable propagation behavior. This study investigates the applicability of a site-specific modeling approach, originally developed for urban street canyons, to characterize peer-to-peer (P2P) IQRF links operating at 868 MHz in typical indoor scenarios, including line-of-sight (LoS), one-turn, and two-turn non-line-of-sight (NLoS) configurations. The received signal powers are compared with well-known empirical models, including international telecommunication union radio communication sector (ITU-R) P.1238-9 and WINNER II, and ray-tracing simulations. The results show that while ITU-R P.1238-9 achieves lower prediction error under LoS conditions with a root mean square error (RMSE) of 5.694 dB, the site-specific approach achieves substantially higher accuracy in NLoS scenarios, maintaining RMSE values below 3.9 dB for one- and two-turn links. Furthermore, ray-tracing simulations exhibited notably larger deviations, with RMSE values ranging from 7.522 dB to 16.267 dB and lower correlation with measurements. These results demonstrate the potential of site-specific modeling to provide practical, computationally efficient, and accurate insights for IQRF network deployment planning in smart building environments.

## 1. Introduction

In recent years, demand for smart buildings has increased due to their potential to improve safety, comfort, and energy efficiency [1]. Wireless sensor networks (WSNs)-based internet of things (IoT) applications have gained widespread attention owing to their major role in enabling smart building functionalities [2]. However, integrating WSN-based IoT applications requires careful consideration of trade-offs among power consumption, deployment cost, and coverage provided by the IoT technology [3]. Particularly, for large-scale deployments in an indoor environment, common wireless communication technologies, such as Wi-Fi, Bluetooth Low-Energy (BLE), and XBee/ZigBee, may face such limitations, which constrain their applicability and efficiency [4]. To address this issue, Long Range Wide Area Network (LoRaWAN) has emerged to offer low power consumption and long-range communication [5]. Nevertheless, the network topologies employed by LoRaWAN may lead to increased deployment costs. Recently, Intelligent Connectivity using Radio Frequency (IQRF) has emerged as an alternative technology for indoor applications due to its low power consumption, cost-effectiveness, mesh networking support, and relatively wide coverage at sub-GHz frequencies [6,7]. These characteristics suggest that IQRF can be particularly suitable for large-scale sensor deployments in buildings where reliable indoor connectivity and low operational cost are required.

As the adoption of IQRF technology expands for sub-GHz IoT applications, accurate and environment-specific propagation modeling becomes crucial to ensure reliable indoor connectivity, efficient node placement, and robust network planning in IQRF-based wireless sensor networks. However, the indoor performance and modeling aspects of IQRF-based wireless networks remain limited in the literature [8,9,10]. In [8], the performance of IQRF in terms of range, network latency, and convergence was evaluated in both indoor and outdoor environments, confirming stable communication within multi-wall rooms. Other experimental study investigated the effect of transmission power on network stability in multi-floor buildings [9]. Moreover, a preliminary study on IQRF indoor propagation analyzed path loss characteristics in corridor environments using peer-to-peer links by comparing measured results with empirical models, such as the international telecommunication union radio communication sector (ITU-R) and wireless world initiative new radio phase II (WINNER II) model [10]. However, there remains a research gap in accurately modeling IQRF propagation behavior in complex indoor environments, particularly those with multiple turns, wall blockages, and non-line-of-sight (NLoS) conditions typical of large building layouts. Existing IQRF studies mainly focus on empirical observations or modeling for a thorough understanding of propagation impairments, such as path loss (PL), affecting the quality and reliability of wireless links [11].

Empirical models offer a simpler and faster way to estimate PL by using measured signal data. This makes them more suitable for network planning and performance evaluation in real environments. Yet, their general formulations often fail to represent the intricate multipath and diffraction effects found in multi-corridor or narrow indoor layouts [12]. Alternatively, ray-tracing and deterministic modeling techniques can be used to provide accurate characterization of indoor propagation. However, they require detailed knowledge of building geometry, material properties, and complex computational setups, which limit their practicality for large-scale IoT or WSN deployments [13,14]. To overcome these limitations, site-specific propagation models may provide a balance between empirical simplicity and geometric precision. These models integrate key structural information, such as wall positions, corridor widths, and turns, while maintaining manageable computational complexity for real-time estimation [15,16,17]. Yet, it is important to note that such modeling approaches have not yet been investigated for the effective planning and deployment of IQRF links in the context of IoT-based smart buildings.

Therefore, in this study, we adapt site-specific propagation models, originally developed for urban street canyons [17], to characterize IQRF peer-to-peer (P2P) communication links in indoor environments. The models are primarily based on diffraction and two-ray ground-reflection principles. They provide an accurate yet computationally efficient approach for estimating received power under both LoS and NLoS conditions, while explicitly accounting for the effects of corridor turns, wall blockages, and diffractions through a limited set of configurable parameters. The performance of the models were evaluated using real-world measurements using IQRF transceivers (TRs) operating at 868 MHz across four representative indoor scenarios: LoS, one-turn NLoS, and two-turn NLoS configurations. In the LoS scenario, the transmitter (Tx) and receiver (Rx) were positioned to directly face each other along a straight corridor. In the one-turn scenario, a wall between two intersecting corridors was introduced, creating a blockage between the TRs. In the two-turn scenario, the Tx and Rx were placed in parallel corridors separated by two walls, and the signal was diffracted through two edges. The one-turn and two-turn configurations were examined following L-shaped, U-shaped, and Z-shaped routes. For all scenarios, theoretical predictions were compared with both measured values and simulation results. Additionally, the performance of the well-known empirical models, including ITU-R P.1238-9 and WINNER II models, was assessed. To the best of the authors’ knowledge, this study presents the first comprehensive P2P performance analysis of IQRF links under highly probable indoor configurations involving multiple turns. Furthermore, for the first time in the literature, the received power prediction performance of models originally proposed for urban street canyons is examined for indoor environments in the 868 MHz band. The results suggest that the site-specific models may provide better performance in one-turn and two-turn scenarios in typical indoor corridor environments when compared to well-known empirical models.

The remainder of the paper is organized as follows. Section 2 addresses the empirical indoor propagation models. Section 3 introduces the proposed modified diffraction-based two-ray model. In Section 4, the implementation details of the study and measurement campaigns are outlined. The findings and analyses are presented in Section 5. Section 6 provides discussion and future work, and Section 7 draws conclusions.

## 2. Empirical Indoor Propagation Models

Wireless communication systems extensively utilize empirical propagation models to assess signal attenuation over distance based on measured data. Typically, these models are derived from extensive field measurements in specific environments. When there are limited computational resources and environmental details, their simplicity and ease of use make them essential for system planning, network optimization, performance analysis, and signal coverage applications. Empirical models such as ITU-R P.1238-9 and WINNER II were found to be effective in predicting received signal strength in indoor IQRF-based communication scenarios [10]. Motivated by these results, these two models are included in this work for comparative evaluation against the proposed site-specific approach. The following sections briefly discuss the ITU-R P.1238-9 and WINNER II empirical PL models.

### 2.1. ITU-R P.1238-9 Model

ITU-R P.1238-9 indoor propagation model estimates PL considering different environmental conditions, such as losses due to multiple floors, transmission through walls. This model is defined for indoor applications with 300 MHz to 100 GHz frequency range. The mathematical expression of PL can be found below [18].

(1) $$L_{ITU}(R)\;[dB]=20log\left(f\right)-28+Nlog\left(\frac{R}{R_{0}}\right)+L_{f\;}\;$$

where f is the frequency in MHz, R is the distance between Tx and Rx in meters,$\;R_{0}$ is the reference distance in meter, N is the distance power loss coefficient, and $L_{f}$ is the floor penetration loss. In our study, all of the measurements were taken on the same floor. Therefore, $L_{f}$ is neglected. Here, N is chosen as 33 since its performance is found to be better than other configurations [10].

### 2.2. WINNER II Model

The WINNER II propagation model is an empirical PL model aiming to evaluate radio channel characteristics for both indoor and outdoor wireless systems. It is based on a series of measurement campaigns carried out under various conditions, including both LoS and NLoS scenarios. One of the important features of this model is that it uses the same modeling approach for different environments, making it flexible and consistent. In this study, the indoor version of the WINNER II model is used to estimate the received power. The mathematical expression of the PL is given as follows [19]

(2) $$L_{WINNERII}(R)\;[dB]=Alog\left(R\right)+B+Clog\left(\frac{f}{5}\right)+X$$

where f is the frequency in GHz, R is the distance between Tx and Rx in meters, A is the PL exponent, B defines the intercept, C is a PL frequency dependence parameter, and X is a parameter specific to NLoS for a corridor room and heavy walls environment. In Table 1, the parameters of the WINNER II model for indoor propagation are listed [10]. The value of X is determined by considering the number of heavy walls $n_{W}$.

It should be noted that both the ITU-R P.1238-9 and WINNER II models are originally formulated in terms of PL. In this study, they are expressed in terms of received power to allow a direct comparison with the measurement data. The conversion from PL to received power is given by the following equation:

(3) $$P_{R}=\frac{P_{T}G_{T}G_{R}}{L_{MODEL}}$$

where $P_{R}$ is the received power, $P_{T}$ is the transmitted power, $G_{T}$ and $G_{R}$ are the Tx and Rx antenna gains, respectively, and $L_{MODEL}$ is the PL for the models given in Equations (1) and (2).

## 3. Site-Specific Models: LoS and NLoS Links

Site-specific propagation models have been extensively used to characterize radio communication in urban street canyon environments, where signal behavior is strongly affected by diffraction and reflection from surrounding structures [17,20,21]. However, the these studies have mainly focused on higher frequency bands (above 2 GHz) and outdoor environments, where propagation characteristics may differ significantly from those observed in sub-GHz indoor settings [22]. In particular, the 868 MHz band, widely used for low-power IoT applications, has received limited attention regarding the site-specific modeling of received power in indoor corridor environments.

In this study, we adopt and adapt the simplified site-specific models presented in [10], which were originally proposed for urban street canyon configurations, due to their reduced computational complexity and minimal environmental requirements compared to more complex deterministic approaches. This adaptation aims to enable simple yet accurate deployment planning of IQRF P2P links operating at 868 MHz in indoor environments, thereby supporting practical IoT-based smart building applications.

In [10], a two-ray-based approach was used for predicting the small-area average received power under both LoS and NLoS conditions, effectively capturing long-term fading and distance-dependent loss. Within this framework, three primary link configurations were defined: (a) LoS, (b) one-turn NLoS, and (c) two-turn NLoS. The following subsections present the background of these configurations and their adaptation to typical indoor corridor environments. It should be noted that these configurations were predefined to represent typical deployment geometries, and the objective of this study is to evaluate model performance under these representative conditions rather than to determine the scenario automatically.

### 3.1. LoS Link

**Figure 1:**
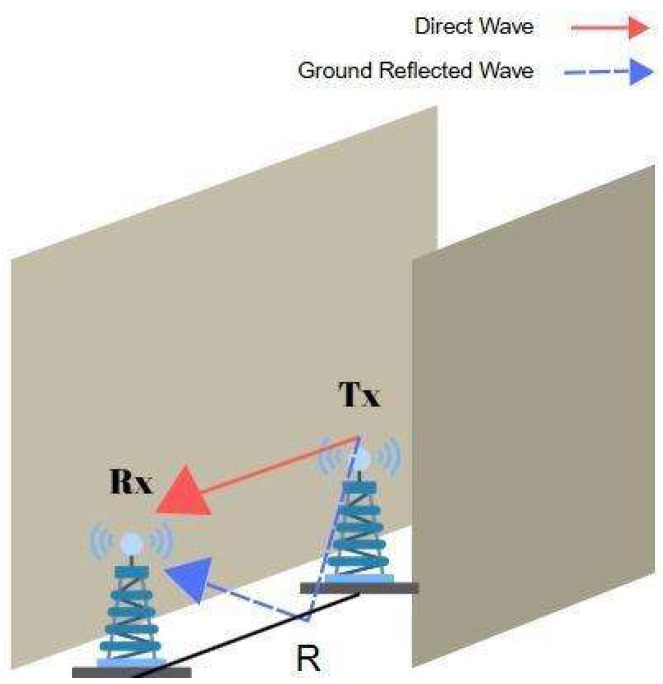
Direct and reflected waves in LoS scenario.

A LoS link occurs when the TRs can directly see each other in the same corridor with walls on either side as shown in Figure 1. In indoor LoS scenarios, reflection plays a significant role, as the signal energy is often conveyed not only through the direct path but also via reflections from the floor, ceiling, or walls.

The distance dependency of the received power in LoS can be modeled using the two-ray ground-reflection model, which integrates direct and ground-reflected paths to estimate the received power [15]. Then, the received power in watts can be found by [23]

(4) $$P_{2-Ray}\left(R\right)=P_{T}{\left(\frac{\lambda }{4\pi }\right)}^{2}|\frac{exp(-\frac{j2\pi \sqrt{R^{2}+{\left(h_{T}-h_{R}\right)}^{2}}}{\lambda })}{\sqrt{R^{2}+{\left(h_{T}-h_{R}\right)}^{2}}}+\Gamma \left(\theta \right)\frac{exp(-\frac{j2\pi \sqrt{R^{2}+{\left(h_{T}+h_{R}\right)}^{2}}}{\lambda })}{\sqrt{R^{2}+{\left(h_{T}+h_{R}\right)}^{2}}}\;{|}^{2}$$

where R is the distance between Tx and Rx antennas, λ is the wavelength, $h_{T}$ is the Tx antenna height, and $h_{R}$ is the Rx antenna height, all in meters. $P_{T}$ is the transmitted power and $\Gamma \left(\theta \right)$ indicates the reflection coefficient. The reflection coefficient, which depends on the incident angle and polarization, is given by [15]

(5) $$\Gamma \left(\theta \right)=\frac{cos\left(\theta \right)-\alpha \sqrt{\epsilon_{R}-\theta \;\;}}{cos\left(\theta \right)+\alpha \sqrt{\epsilon_{R}-\theta \;}}$$

where θ = 90°−α, α is the incident angle, α $=1/\epsilon_{R}$ or 1 for vertical or horizontal polarization, respectively, and $\epsilon_{R}$ represents the relative dielectric constant. The electromagnetic properties of the reflecting surface significantly influence the reflection behavior. For this reason, $\epsilon_{R}$ is included in the computation of the reflection coefficient. In addition, α plays a critical role in determining the magnitude and phase of the reflected wave, especially in scenarios involving long-range propagation.

In this study, the antenna gain is explicitly included in the received power calculations, as the measurement setup has external antennas. Therefore, the received power was calculated by incorporating both Tx and Rx antenna gains into the propagation models as follows

(6) $$P_{R}\left(R\right)=P_{2-Ray}\left(R\right)G_{T}G_{R}$$

where $G_{R}$ is Rx antenna gain and $G_{T}$ is Tx antenna gain.

### 3.2. One-Turn NLoS Link

A one-turn NLoS link occurs when the Tx and Rx are located in two corridors that intersect each other perpendicularly, which constitutes an L-shaped path. In indoor environments, i.e., intersecting corridors of a building, emitted radio signals can reach the Rx located in another corridor by turning a corner. When there is an obstacle between devices, diffraction becomes a dominant propagation path, enabling the signal to bend around corners or edges. However, both reflected and diffracted waves lose amplitude due to energy dispersion and material interaction.

Signal propagation in indoor one-turn scenarios, where a radio signal must go from one corridor to a perpendicular corridor, is primarily influenced by reflection, diffraction, and scattering. Reflected rays near the corner rapidly lose power due to relatively normal incidence and are unable to propagate farther into the side corridor. Despite undergoing significant loss at the turning point, diffracted signals have less distance dependency and can travel farther. Scattering from objects such as door handles or fire equipment also helps to disperse signals in numerous directions. Overall, diffraction plays a major role in maintaining signal continuity in NLoS indoor scenarios.

**Figure 2:**
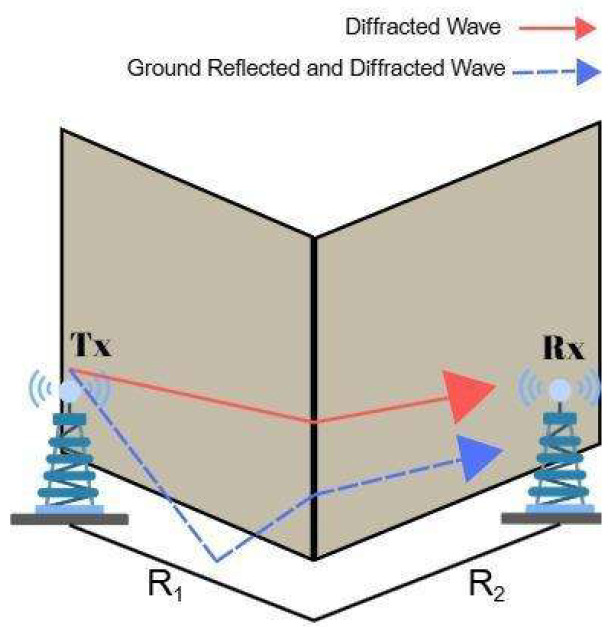
Reflected and diffracted waves in one-turn scenario.

Figure 2 illustrates a representative one-turn NLoS scenario, where the signal reaches the Rx after undergoing both ground reflection and corner diffraction following a single turn in the corridor layout.

In the one-turn scenario, the model adds the diffraction to Equation (6). If the total distance between Tx and Rx is significantly greater than the antenna heights, the diffraction becomes the same for both rays. Therefore, the diffraction coefficient $K_{D}$ is added to the formulation as a loss, and the one-turn received power becomes [17]

(7) $$P_{1-Turn}=P_{R}\left(R_{1}+R_{2}\right)\frac{|K_{D}{|}^{2}}{R_{1}R_{2}}(R_{1}+R_{2})$$

where $P_{R}$ is the received power given Equation (6) for the distance $R_{1}+R_{2}$. For long distances, $K_{D}$ will be assumed as a constant with the diffraction angle of nearly 90°.

### 3.3. Two-Turn NLoS Link

**Figure 3:**
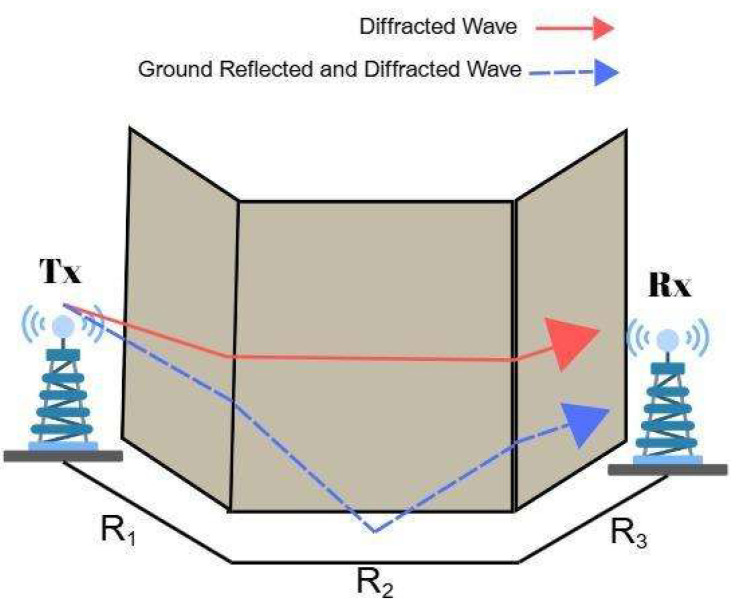
Reflected and diffracted waves in a two-turn scenario.

In indoor corridor environments, the transmitted signal may reach the Rx by undergoing two successive turns, which constitute U-shaped or Z-shaped routes as shown in Figure 3. When the Tx and Rx are positioned in two separate parallel corridors and there is another corridor intersecting them perpendicularly, the radio signal should turn two corners to complete the link. In these scenarios, each significant propagation path experiences two diffraction events, typically occurring at the corners of the walls. This will result in additional attenuation compared to the one-turn configuration. Since one-turn links already cause a significant amplitude loss, other links beyond two turns are neglected [24].

In the two-turn scenario, the model inserts another diffraction parameter $D_{2}$ into Equation (7). The diffraction parameter will be the same for both rays as in the one-turn scenario. Therefore, the two-turn received power becomes [17]

(8) $$P_{2-Turn}=P_{R}(R_{1}+R_{2}+R_{3})\frac{|K_{D1}K_{D2}{|}^{2}}{R_{1}R_{2}R_{3}}(R_{1}+R_{2}+R_{3})$$

where $K_{D1}$ and $K_{D2}$ are the diffraction coefficients of one-turn and two-turn, respectively. $K_{D2}$ parameter will also be constant as mentioned before, assuming 90° of diffraction angle.

## 4. Implementation

This section presents the implementation details of the site-specific models for P2P IQRF communication links in indoor corridor environments. The main objective was to evaluate the performance of the models to accurately predict received power across distinct geometrical configurations that are commonly encountered in real building layouts.

In [10], a preliminary study was presented considering only two simple propagation conditions, LoS and one-turn NLoS, to explore the feasibility of empirical modeling for P2P IQRF communication at 868 MHz. In this study, we extend this framework by incorporating two-turn NLoS scenarios configurations to better reflect the indoor corridor propagation scenarios, where the transmitted signal undergoes two successive diffractions before reaching the Rx. Such two-turn conditions, while previously analyzed only in urban street-canyon contexts, are in fact frequently encountered in multi-corridor indoor environments such as office buildings, hospitals, or university campuses.

Measurement campaigns were conducted at the Engineering Faculty of Atılım University (Ankara, Türkiye). Each measurement scenario was designed to correspond to the theoretical link types described in Section 3: (a) a straight corridor (LoS link) scenario, (b) an L-shaped (one-turn NLoS link) scenario, (c) a U-shaped (two-turns NLoS link) scenario, and (d) Z-shaped (two-turn NLoS link) scenario. Measurements were repeated across multiple corridors (four for LoS and two for each NLoS configuration), ensuring that the derived parameters represent a range of realistic indoor conditions rather than a single environment. The results presented in this study are the averaged values of these repetitions. The details of the measurement setup and each scenario are described in the following subsections.

### 4.1. Measurement Setup

The measurements were conducted using IQRF TRs (TR-72DCT, IQRF Tech, Jičín, Czech Republic) with external straight-line dipole antennas (SLDAs) [25]. One of the TRs was configured as a Tx and the other was configured as an Rx. For practicality, the Tx platform was mobile during all measurements, and the Rx platform remained fixed. Both Tx and Rx antennas were positioned at a height of 1.6 m above the ground. A P2P topology is employed during the data collection procedures to construct a network.

**Figure 4:**
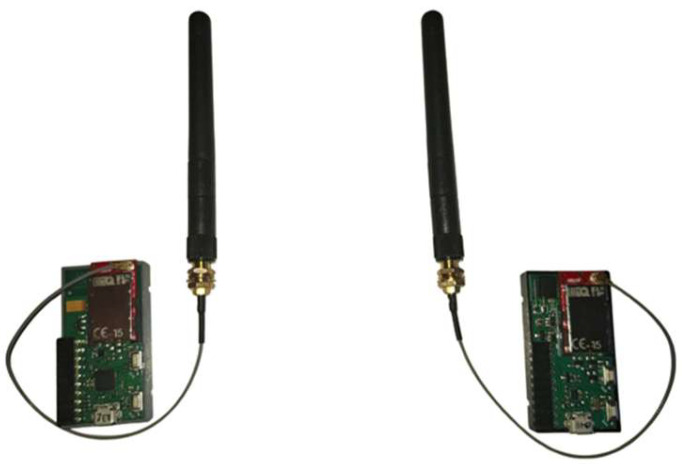
IQRF TRs with external antenna.

At each measurement point, a total of 3000 data packets were sent and at Rx, the received signal strength indicator (RSSI) value was recorded for each individual packet. The RSSI value obtained at a measurement point was calculated as the average RSSI value of these 3000 packets, and all measurements were performed at 868 MHz. The IQRF TRs are shown in Figure 4. In addition, the configurations for the IQRF TRs can be found in Table 2.

### 4.2. First Scenario: LoS Link

**Figure 5:**
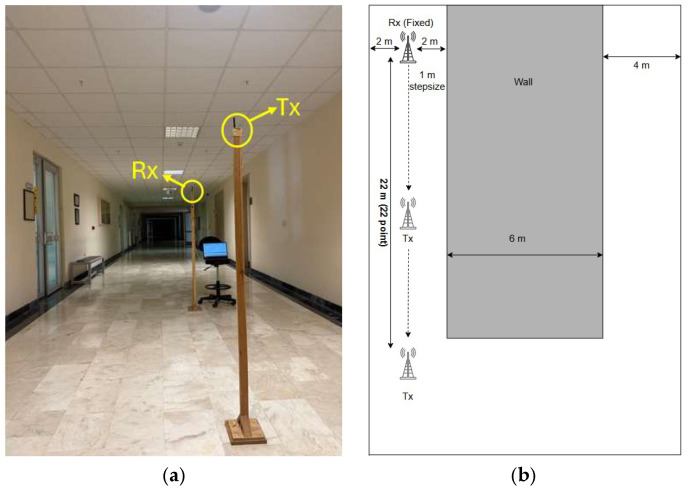
Indoor LoS scenario. (**a**) Photograph of the measurement environment, (**b**) Top view of the corridor layout for LoS scenario.

This scenario assumes that the Tx and Rx are positioned within the same linear segment of a building’s corridor. 22 measurements were performed with 1 m intervals along a straight corridor, with the distance between the Tx and Rx ranging from 1 to 22 m. Figure 5 illustrates the positions of the Tx and Rx along with the measurement environment.

### 4.3. Second Scenario: L-Shaped NLoS Link

**Figure 6:**
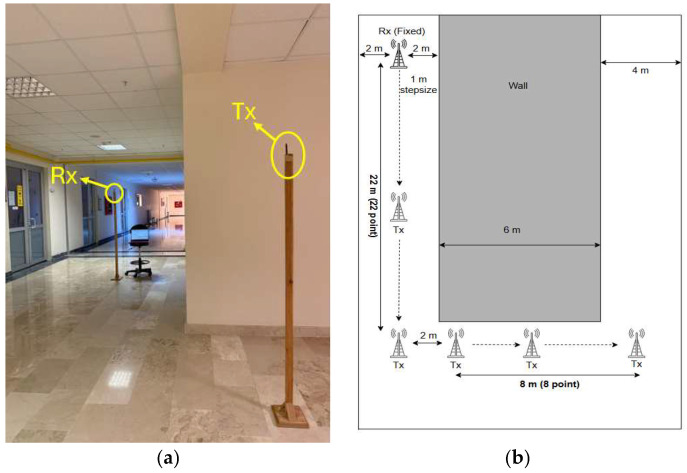
Indoor L-shaped NLoS scenario. (**a**) Photograph of the measurement environment, (**b**) Top view of the corridor layout for the L-shaped NLoS scenario.

In the second scenario, Tx and Rx are positioned in the corridors that intersect one another, reflecting the L-shaped corridor geometries that exist frequently in interior settings, as illustrated in Figure 6. This configuration allows an evaluation of signal propagation behavior as the signal turns a corner around the wall. After the 22 m LoS measurements, a single 90° turn was made, and 8 measurement points were selected along 8 m and measurements were taken at 1 m intervals.

### 4.4. Third Scenario: U-Shaped NLoS Link

**Figure 7:**
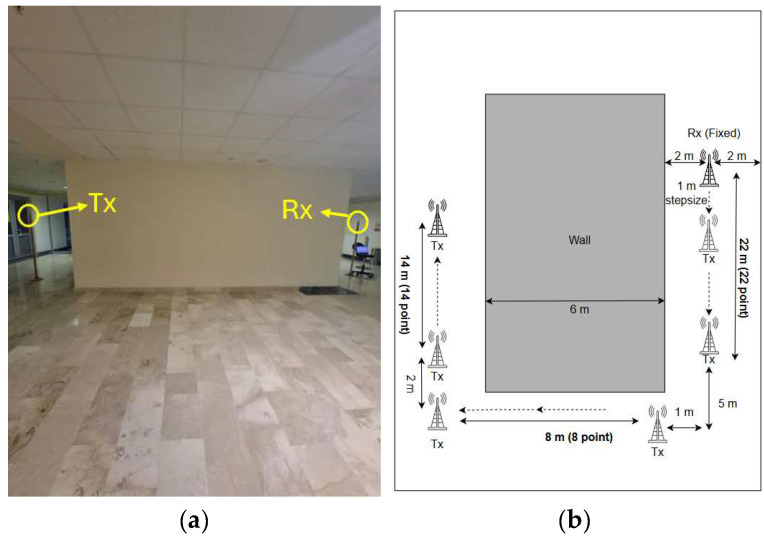
Indoor U-shaped NLoS scenario. (**a**) Photograph of the measurement environment, (**b**) Top view of the corridor layout for U-shaped NLoS scenario.

The third scenario focuses on a propagation path, involving two diffraction events occurring at two different wall corners, forming a U-shaped path. This configuration enables a more thorough analysis of the effects of several sharp-angle diffractions on the received signal. While the first vertical segments have 22 measurement points covering 22 m, the second vertical segment has 14 measurement points. The horizontal segment has 8 measurement points spread throughout a 1 m interval with a total distance of 9 m. The modeling of the third scenario can be found in Figure 7.

### 4.5. Fourth Scenario: Z-Shaped NLoS Link

**Figure 8:**
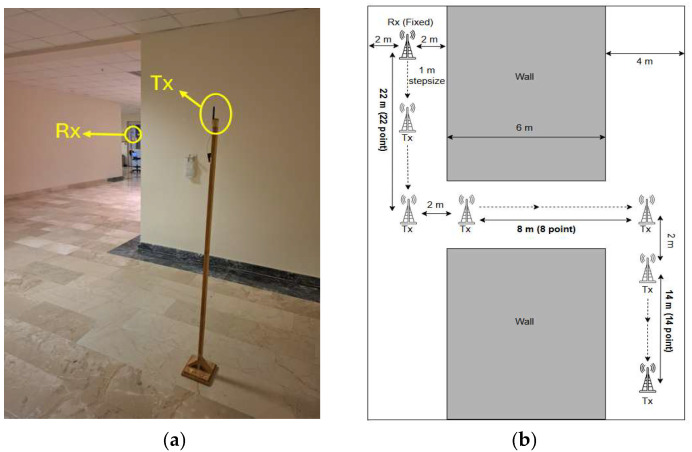
Indoor Z-shaped NLoS scenario. (**a**) Photograph of the measurement environment, (**b**) Top view of the corridor layout for the Z-shaped NLoS scenario.

The last scenario investigates a propagation channel characterized by two diffraction occurrences at diagonally opposite wall corners. Initially, 22 measurements were collected at 1 m intervals along a straight LoS section covering 22 m. Next, Rx was shifted laterally by 10 m with 1 m spacing to obtain a 90° turn, resulting in an additional 8 measurements. Finally, a further 14 measurements were taken in the forward direction after the second 90° turn, again at 1 m intervals, to complete the Z-shaped path. The model of the Z-shaped NLoS link can be found in Figure 8.

## 5. Results

To provide a comprehensive evaluation of the site-specific modeling approach, two experimental cases were examined under the considered scenarios. The first experimental case included LoS, L-shaped NLoS, and U-shaped NLoS links, while the second experimental case consisted of LoS, L-shaped NLoS, and Z-shaped NLoS links. The measured data were compared with the corresponding site-specific models described in Section 3, simulation results, and well-known empirical models introduced in Section 2. Furthermore, the correlation coefficient (*ρ*) and root mean square error (RMSE) metrics were calculated for each scenario to quantitatively assess the agreement between the measured and predicted received power values. Notably, RMSE, defined as the square root of the average squared deviation between predicted and measured values, indicates the overall prediction error, while the correlation factor represents the degree of linear relationship between them [26]. In accordance with conventional evaluation methodologies used in propagation modeling, the RMSE values were calculated within the logarithmic (dB) domain by comparing the measured and predicted received power levels, both expressed in dB [27]. The detailed analysis of the achieved results is presented in the following subsections.

### 5.1. Comparison with Measurements

To ensure the validity of the site-specific modeling, first, the measured data with the theoretical predictions provided by the models were compared. This comparison aims to assess how accurately the site-specific models capture the propagation characteristics observed in practical indoor environments under both LoS and NLoS conditions.

Before the comparisons, diffraction coefficients were estimated by fitting the models to the measurement data using the nonlinear least-squares method, which is widely applied in wireless propagation modeling to fit empirical or site-specific model parameters to experimental observations. In the L-shaped NLoS scenario, a single diffraction coefficient provided consistent agreement with the measurements due to the identical geometric layout. However, for the two-turn scenarios (U-shaped and Z-shaped NLoS links), two distinct diffraction coefficients were required to achieve higher accuracy. The Z-shaped NLoS configuration involves two different walls, each introducing separate diffraction characteristics, whereas in the U-shaped case both diffraction points are located along the same wall. Therefore, scenario-dependent diffraction coefficients were determined to better represent the observed propagation behavior. The diffraction coefficients determined for each link are listed in Table 3.

**Figure 9:**
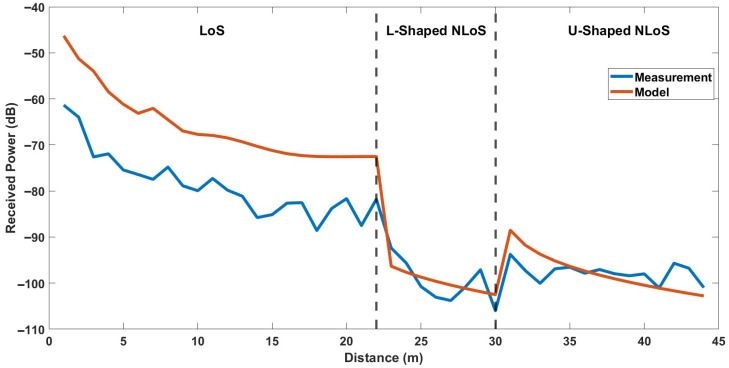
Comparison of the site-specific modeling and measurements for LoS, L-Shaped NLoS and U-Shaped NLoS links.

**Figure 10:**
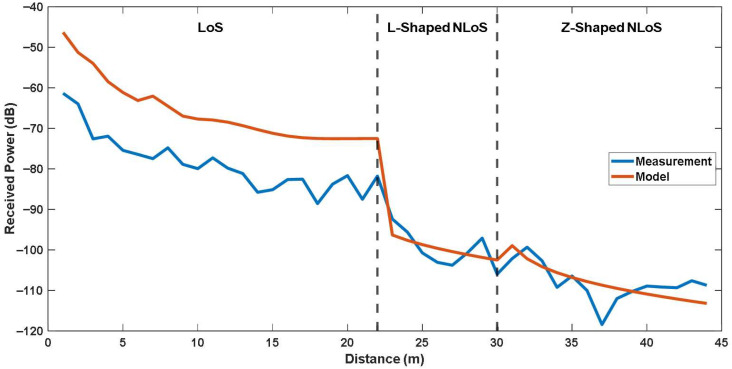
Comparison of the site-specific modeling and measurements for LoS, L-Shaped NLoS and Z-Shaped NLoS links.

For the first experimental case, including LoS, L-shaped NLoS, and U-shaped NLoS links, the comparison of measured and calculated received powers are shown in Figure 9. Moreover, for the second experimental case, including LoS, L-shaped NLoS, and Z-shaped NLoS links, the comparison of measured and calculated received powers are shown in Figure 10. It should be noted that the x-axis represents the distance between the Tx and Rx, which corresponds to the total path length defined as the sum of $R_{1}$, $R_{2},$ and $R_{3}$. The vertical dashed lines mark the transition points between different propagation links.

As shown in Figure 9 and Figure 10, the received power exhibits a consistent decreasing trend as the total propagation distance increases, which aligns with conventional propagation behavior. The models accurately capture the overall attenuation trend of the received signal along with the certain peaks of the measurements, which may be caused by reflections and diffractions. Overall, although the site-specific modeling shows compatible results with the measured data, especially for NLoS links, noticeable differences are observed at LoS link. To quantitatively evaluate these differences, both the RMSE and *ρ* were calculated for each experimental scenario. The results are also listed in Table 3.

As can be seen from Table 3, the model yields RMSE values of 13.002 dB for LoS link, 3.199 dB for L-shaped NLoS link, 3.588 dB for U-shaped NLoS link, and 3.801 dB for Z-shaped NLoS link, with corresponding correlation factors of 0.944, 0.723, 0.469, and 0.607, respectively. Obviously, the highest RMSE occurs in the LoS scenarios, which may be attributed to environmental irregularities in indoor scenarios. Unexpected scattering or reflection effects that are not taken into consideration in the proposed model may be introduced. Contrary to the outdoor scenarios, in an indoor environment two-ray ground-reflection model may remain insufficient for estimating the received signal power since there will be additional reflections caused by the side walls and ceilings.

On the other hand, the lowest RMSE is observed in the L-shaped NLoS links. The correlation factor also shows consistency in L-shaped NLoS link, which means the site-specific modeling accurately adds the diffraction losses to the calculation. For the U-shaped NLoS link, the RMSE indicates a low prediction error in terms of received power levels, despite a low correlation factor. It means that while the model approximates the average received power well, it does not fully capture the local variations along the propagation path. In the Z-shaped NLoS link, the RMSE value is still quite adequate with an increased correlation factor. In addition, $K_{D2}$ value for the Z-shaped scenario was estimated lower than the U-shaped scenario. This indicates that there are more diffraction losses in the Z-shaped NLoS link, as expected for two independent corner turns.

In summary, the proposed site-specific modeling approach, although simplified to a two-ray structure, provides a robust and accurate representation of the signal behavior in NLoS conditions, where diffraction and reflection effects dominate. Its ability to capture power variations around corners and maintain continuity across transitions highlights its suitability for realistic indoor environments with obstructed paths. For LoS links, despite the high correlation factor indicating general consistency, the model shows limited capability in representing the richer multipath reflections typical of open corridors. Therefore, the site-specific modeling approach is particularly advantageous and better suited for NLoS scenarios, where geometric constraints and diffraction effects are the primary determinants of propagation behavior.

**Figure 11:**
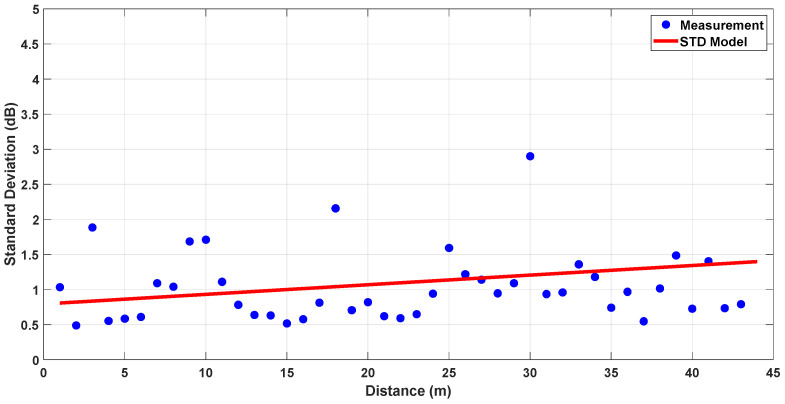
Standard deviation model for LoS, L-Shaped NLoS and U-Shaped NLoS links.

**Figure 12:**
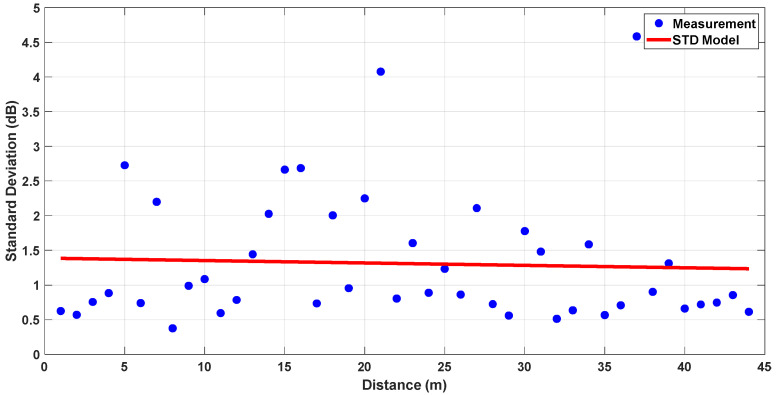
Standard deviation model for LoS, L-Shaped NLoS and Z-Shaped NLoS links.

To further support the reliability of the measurement results, the standard deviation of measurements is calculated using the recorded raw data. As 3000 packets were transmitted at each location, 3000 individual received power values were available. Furthermore, four different measurements were taken in different corridors, allowing for a reliable estimation of the statistical distribution. The standard deviation was computed directly from these samples. To model the variation in standard deviation with respect to distance, linear regression was applied to the calculated values. For both cases, obtained standard deviation (STD) models can be found in Figure 11 and Figure 12.

The results indicate that the standard deviation does not significantly change with distance. The regression-based model fits the measurement data reasonably well, with standard deviation values generally confined to around 1 dB. This implies that environmental factors caused limited variation in the signal, and the channel can be considered statistically consistent and suitable for reliable modeling.

### 5.2. Comparison with 3D Ray-Tracing Simulations

To further assess the predictive accuracy and practical applicability of the proposed site-specific modeling approach, a detailed comparative analysis was performed against both experimental measurements and 3D ray-tracing simulation results. In this context, simulations were performed using the X3D 3D ray-tracing model in Wireless InSite software (release 4.0) [28]. The ray-tracing model considered a large number of propagation mechanisms, including reflections and diffractions from the corridor walls, edges, ceiling, and floor. In our configuration, the model traced up to 25 rays per transmitter–receiver pair. However, the actual number of valid rays reaching the receiver depends on the geometry, signal power, and the collection radius of the receiver sphere defined in the X3D method.

**Figure 13:**
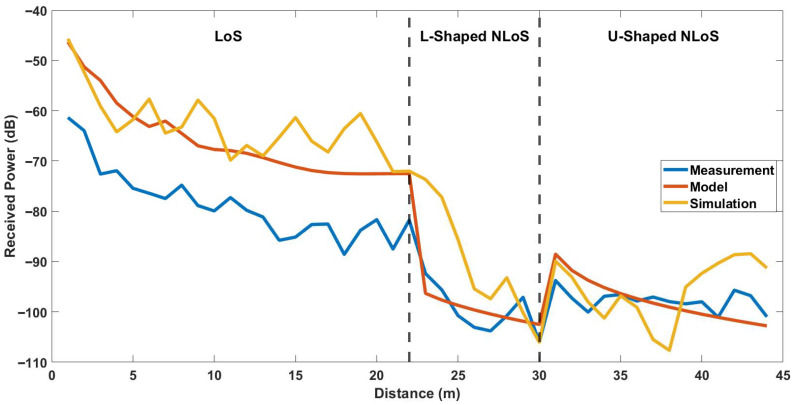
Comparison of the site-specific modeling, simulation and measurements for LoS, L-Shaped NLoS and U-Shaped NLoS links.

**Figure 14:**
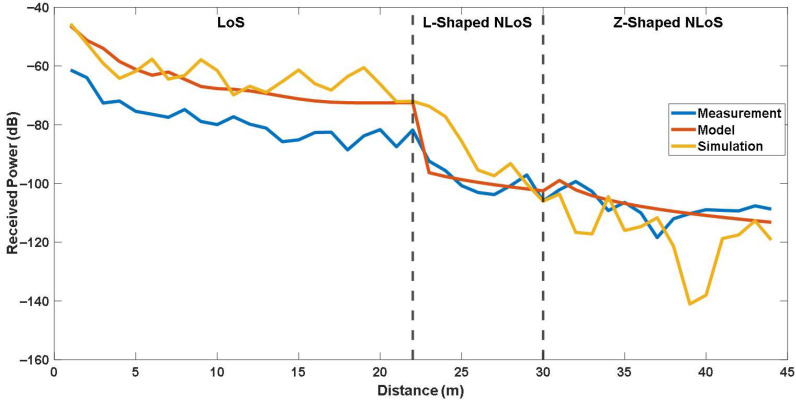
Comparison of the site-specific modeling, simulation and measurements for LoS, L-Shaped NLoS and Z-Shaped NLoS links.

Figure 13 and Figure 14 present a comparison of the received signal power obtained from measurements, the site-specific modeling, and the ray-tracing simulations for the first and second experimental case, respectively. As shown in the figures, the simulated results exhibit larger fluctuations compared with the measured data. For a quantitative comparison, the error metrics calculated between the simulation and the model for each link are provided in Table 4. As can be seen from the table, The simulation yielded RMSE values of 16.267 dB for LoS link, 11.634 dB for L-shaped NLoS link, 7.522 dB for U-shaped NLoS link, and 13.975 dB for Z-shaped NLoS link, with corresponding correlation factors of 0.717, 0.805, 0.050, and 0.160, respectively. The largest deviation is observed in the LoS scenario, where the ray-tracing simulation exhibits a significantly higher RMSE. The results also indicate that while the L-shaped NLoS scenario shows moderate correlation, the U-shaped and Z-shaped links suffer from poor agreement between simulated and measured results.

It should be noted that the primary purpose of including the ray-tracing simulations in this study was not to reproduce the exact measured power levels. It was aimed to illustrate the relative prediction behavior of a full 3D ray-tracing approach compared to the proposed site-specific model under identical geometric configurations. While the simulator provides a comprehensive electromagnetic environment, its accuracy is highly sensitive to the precise characterization of materials and geometrical features. In practice, small uncertainties in the corridor dimensions, wall thickness, or dielectric parameters can lead to notable differences between simulated and measured power levels, especially in confined indoor corridors with multiple reflection paths. In this study, all geometric and material parameters were defined according to available site information. Thus, some mismatch between simulation and measurement is expected. Nevertheless, a more detailed site characterization, including precise material permittivity measurements and fine-grained geometric modeling, could further improve the agreement between simulation and experimental results in future studies.

### 5.3. Comparison with Empirical Models

To evaluate the performance of the LoS and NLoS models, a comparative analysis was conducted against two empirical indoor propagation models, including ITU-R P.1238-9 and WINNER II, both widely used for radio channel characterization in indoor wireless systems. As can be deduced from Section 2, ITU-R P.1238-9 provides frequency-dependent PL equations for various indoor environments, while the WINNER II model extends this approach by incorporating additional parameters to capture large-scale fading and frequency variability up to 6 GHz. However, both models were primarily developed for typical indoor environments without accounting corridor turns. In addition, they do not explicitly model the diffraction and reflection mechanisms that dominate in multi-corridor layouts. Therefore, although there is an initial attempt in [10], their direct applicability to sub-GHz indoor IoT networks, such as IQRF systems, remains uncertain.

**Figure 15:**
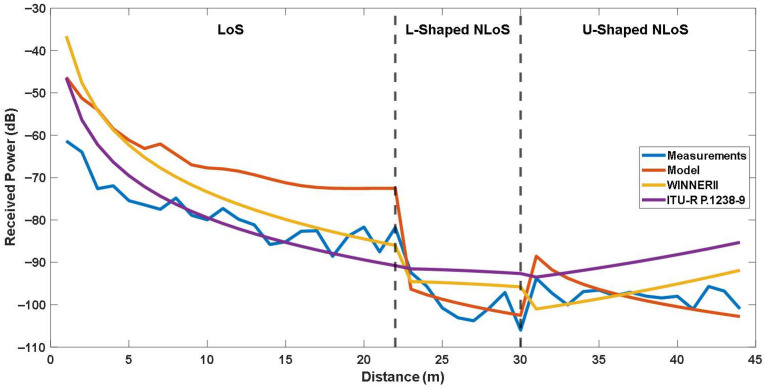
Comparison of the site-specific and empirical models for LoS, L-Shaped NLoS and -Shaped NLoS links.

**Figure 16:**
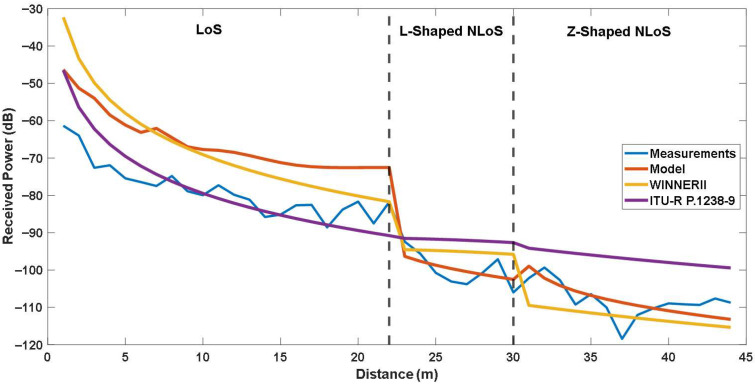
Comparison of the site-specific and empirical models for LoS, L-Shaped NLoS and Z Shaped NLoS links.

To assess these limitations, all models were evaluated under identical conditions using the same measurement data and error metrics. The results are summarized in Table 5, while Figure 15 and Figure 16 illustrate the corresponding received power comparisons across LoS, L-shaped NLoS, U-shaped, and Z-shaped configurations.

In LoS scenarios, all models exhibit a smooth decrease in received power with increasing distance, consistent with theoretical propagation behavior. The ITU-R P.1238-9 model achieves the lowest RMSE of 5.694 dB, slightly outperforming the site-specific model, likely due to its empirical tuning for direct-path propagation conditions.

In contrast, for L-shaped NLoS scenarios, where the signal diffracts around a corner, the site-specific model demonstrates better performance with an RMSE of 3.199 dB, compared to 6.315 dB for WINNER II and 8.901 dB for ITU-R P.1238-9. This improvement stems from the explicit inclusion of diffraction loss and reflection coefficients in the site-specific formulation, enabling better representation of energy decay at turning points.

For U-shaped and Z-shaped NLoS scenarios, which involve two successive turns and represent some of the most challenging propagation cases in indoor corridors, the site-specific model again shows significantly better alignment with the measured data. For U-shaped NLoS, the site-specific model achieves higher accuracy with an RMSE of 3.588 dB, compared to 4.127 dB for WINNER II and 8.917 dB for ITU-R P.1238-9. In addition, For Z-shaped NLoS, the site-specific model yields better performance with an RMSE of 3.801 dB, compared to 5.850 dB for WINNER II and 11.905 dB for ITU-R P.1238-9. As shown in Figure 15 and Figure 16, it maintains a consistent attenuation trend that closely follows the measurements, while empirical models deviate due to their inability to account for multiple diffraction events. The results suggest that site-specific modeling may provide a more accurate yet computationally feasible approach for complex indoor geometries.

Overall, these findings demonstrate that while empirical models like ITU-R P.1238-9 and WINNER II are suitable for general-purpose indoor analysis, they are less effective in geometrically constrained sub-GHz multi-corridor environments. The site-specific modeling successfully bridges this gap by adapting diffraction-based two-ray mechanisms to IQRF P2P links, achieving the best overall balance between accuracy and simplicity across all evaluated scenarios.

## 6. Discussion

### 6.1. Limitations

Although the proposed site-specific modeling approach provides acceptable performance across various indoor corridor scenarios, certain limitations should be considered regarding its generalizability. In particular, for the NloS links, the diffraction coefficients used in this study were empirically derived through nonlinear fitting to minimize the error between the model and the measured data. As such, these coefficients are inherently scenario-specific and reflect the propagation characteristics of the particular test environment, including corridor geometry, wall materials, and antenna placements. As is known, diffraction behavior can vary substantially depending on the incidence angle, surface permittivity, and the spatial configuration between the transmitter, receiver, and diffraction edge. Therefore, applying the experimentally determined coefficients directly to other indoor environments could lead to increased modeling error. This limitation does not invalidate the model itself but rather highlights the context-dependent nature of empirically fitted parameters. Thus, it is important to note that this study does not attempt to derive a physically generalized diffraction formulation. Instead, it focuses on evaluating the feasibility of adapting a previously validated outdoor site-specific model to indoor IQRF-based IoT networks, thereby serving as a proof-of-concept demonstration.

On the other hand, in [17], diffraction was modeled through an effective parameter that combines the diffraction and scattering effects occurring at urban intersections. Although this parameter can be denoted as a diffraction coefficient, it represents an aggregate effect rather than a purely analytical diffraction term, as it implicitly accounts for the geometry, distance-dependent attenuation, and corner-induced scattering. Following a similar basis, this study adapts this formulation to indoor corridor environments, where wave propagation is dominated by corner diffraction and multipath effects within confined geometries. It is important to emphasize that the empirical coefficients derived in this study are not directly equivalent to the theoretical diffraction coefficients. Rather, they represent effective fitting parameters that encapsulate both diffraction and scattering losses within the indoor corridor structure. This approach aligns with the concept introduced in [17], where a single diffraction/scattering parameter was used to represent the cumulative corner-turning effects in urban street canyons.

Another potential limitation is the model’s relatively lower performance in LoS conditions compared with well-known empirical models such as ITU-R P.1238-9 and WINNER II. This difference may be attributed to the simplified handling of multipath effects in the site-specific formulation. In open corridor environments, reflections from surrounding surfaces can become more dominant, introducing variations that are not fully represented by the current model structure.

### 6.2. Future Work

Future studies could be directed to extend the adapted site-specific modeling toward broader applicability and higher physical interpretability. Specifically, its performance in LoS scenarios could be further improved by extending the formulation to include additional dominant reflections, particularly from side walls and ceilings. In addition, integrating deterministic diffraction formulations such as those defined in ITU-R P.526 [29] would allow frequency-dependent and geometrical effects to be more accurately represented. Moreover, conducting additional measurement campaigns in various buildings with different corridor geometries and wall materials could provide valuable insight into the sensitivity and transferability of the empirically derived parameters. Another promising direction involves developing hybrid approaches that combine empirical site-specific modeling with data-driven adaptation techniques, enabling the model to adjust dynamically to changing propagation environments.

Furthermore, in this study, measurements were conducted in multiple corridor environments, including four distinct configurations for the LoS case and two independent corridors for each NLoS scenario (L-shaped, U-shaped, and Z-shaped). In this way, it was aimed to ensure that the derived coefficients reflect a range of realistic indoor conditions rather than a single measurement environment, thereby improving their representativeness within typical building layouts. Nevertheless, a more detailed analytical comparison between empirically derived and theoretical coefficients requires explicit characterization of the diffraction models, including wedge or even knife-edge, incidence angles, and surface electromagnetic properties for each corner. This may provide a valuable direction for future work toward developing a more physically generalized diffraction framework for indoor environments.

## 7. Conclusions

In this study, it was aimed to adapt a site-specific propagation model, originally developed for urban street canyon environments, to characterize indoor radio propagation at 868 MHz for IQRF-based point-to-point links. Two experimental campaigns were conducted under distinct corridor configurations, including LoS, L-shaped NLoS, U-shaped NLoS, and Z-shaped NLoS links. Measurement results were compared with both well-known empirical models, including ITU-R P.1238-9 and WINNER II, and 3D ray-tracing simulations to comprehensively assess prediction accuracy and computational efficiency.

The adapted site-specific model exhibited better performance in all evaluated NLoS scenarios. Specifically, it achieved RMSE values of 3.199 dB, 3.588 dB, and 3.801 dB for the L-shaped, U-shaped, and Z-shaped NLoS links, respectively, outperforming the ITU-R P.1238-9 model, whose corresponding errors exceeded 8.9 dB, and the WINNER II model, which yielded values above 4.1 dB in the same scenarios. Furthermore, ray-tracing simulations showed notably larger deviations, with RMSE values of 16.267 dB, 11.634 dB, 7.522 dB, and 13.975 dB for the LoS, L-shaped, U-shaped, and Z-shaped links, respectively, On the other hand, for the LoS link, ITU-R P.1238-9 achieved the most accurate result, with an RMSE of 5.694 dB. Despite this, the site-specific modeling provided more stable and physically consistent predictions across complex diffraction-dominated NLoS environments.

The results achieved from this study may suggest useful insights on the applicability of the site-specific modeling approach for indoor sub-GHz propagation analysis. The results demonstrated that a method originally presented for outdoor urban canyons can effectively characterize indoor corridor behavior. Consequently, this work may establish a reliable and computationally efficient ground for IQRF network deployment planning and link budgeting in smart building scenarios.

## Figures and Tables

**Table 1:** Parameters for WINNER II model.

Model	A	B	C	X
WINNER II LoS	18.7	46.8	20	-
WINNER II NLoS	36.8	43.8	20	12$\;(n_{w}-1)$

**Table 2:** Measurement setup configurations.

Parameter	Value	Unit
Transmission Frequency	868	MHz
Transmitted Power	10	dBm
Tx Antenna Gain	2.15	dBi
Rx Antenna Gain	2.15	dBi
Tx Antenna Height	1.6	m
Rx Antenna Height	1.6	m

**Table 3:** Error analysis and diffraction values of LoS and NLoS links.

Link	RMSE (dB)	*ρ*	$K_{D1}$	$K_{D2}$
LoS	13.002	0.944	-	-
L-Shaped NLoS	3.199	0.723	0.0735	-
U-Shaped NLoS	3.588	0.469	0.0735	4.9461
Z-Shaped NLoS	3.801	0.607	0.0735	1.6803

**Table 4:** Error analysis of each link for simulation results.

Link	RMSE (dB)	*ρ*
LoS	16.267	0.717
L-Shaped NLoS	11.634	0.805
U-Shaped NLoS	7.522	0.050
Z-Shaped NLoS	13.975	0.160

**Table 5:** Error analysis of each link for site-specific and empirical models.

Model	LoS		L-Shaped NLoS		U-Shaped NLoS		Z-Shaped NLoS	
	RMSE (dB)	*ρ*	RMSE (dB)	*ρ*	RMSE (dB)	*ρ*	RMSE (dB)	*ρ*
Site-Specific	13.002	0.944	3.199	0.723	3.588	0.469	3.801	0.607
ITU-R P.1238-9	5.694	0.943	8.901	0.604	8.917	−0.403	11.905	0.510
WINNER II	9.730	0.943	6.315	0.604	4.127	−0.403	5.850	0.510

## Data Availability

The data presented in this study are available on request from the corresponding author due to ethical restriction.
